# Epidemic of Plague in St. Petersburg, Russia

**Published:** 1865-06

**Authors:** 


### EPIDEMIC OF PLAGUE IN ST. PETERSBURG, RUSSIA.

The newspaper accounts are as follows:—"An epidemic, resembling in its fatality the Asiatic cholera, has for some months devastated the interior of Russia. Apparently taking its origin in Siberia, it has gradually swept down southward, spreading more widely on either side as it advances. As yet it has completely baffled the skill of the Russian physicians, and of those professors of medicine who have proceeded from Germany to study its symptoms. In many respects this epidemic resembles the celebrated plague of Athens, which decimated Attica in the second and third years of the Peloponnessian war. Like it, the epidemic belongs to the class of eruptive typhoid disorders. The person seized immediately despairs of recovery; he loses memory and hope together. Like it, too, the Siberian fever is accompanied generally by a hoarse cough and violent retching, and the victim seldom survives beyond the ninth day. There is some difficulty in obtaining a reliable account of the disease, for the Russian officials, never very communicative, have endeavored to conceal the existence of the disease. But it has touched one or two towns in Austria and Prussia, and rages at St. Petersburg. The deaths in the latter city are acknowledged to amount to eighty or one hundred per day, but it is suspected they are five times as numerous.

---

The disease is said to have assumed a mitigated form in Germany, but very great alarm prevails throughout the continent. Men hoped that with the Asiatic cholera the last great scourge of the human race had passed away, but they suddenly find themselves confronting a pestilence which advances as rapidly as a prairie conflagration, floating on the rivers, and borne on the air. Apprehension, too, as in the case of the Asiatic cholera, predisposes to the disease.

"A plague of this description, raging in St. Petersburg, cannot be long absent from other European capitals. It marches steadily and surely. Already its route is traced by death and mourning, and its future track has been pointed out. In such a case quarantine regulations are nearly useless. No plague was ever yet kept away from our shores by delaying a ship from an infected port at a distance from the harbor. The fever may be conveyed in a letter, a bale of goods, a waif, or straw from the ship wafted to the shores. It may be taken up by the wind passing over the deck, and be borne mysteriously, despite of all precautions, to the crowded town. Physicians may dispute whether it is infectious or contagious. Such discussions may be interesting to the profession, but the practical truth is, that whether a plague be conveyed by the air or by contact, there is no means of staying its progress to any land to which it may be the will of Providence to visit with such a scourge."—*Liverpool Post.*

"TERRIBLE EXTENT OF THE RAVAGES OF THE DISEASE.—The total number of cases has reached ten thousand, of deaths two thousand, and the average number of cases is one hundred a day. No less than forty physicians are reported dead, though whether they have fallen victims to the disease is not stated. The account which the correspondent gives of the disease itself, is that 'it is not cholera, but plague, with dilated pupils, carbuncles, and pestilential bubo.' Dr. Charles Murchison, physician to the London Fever Hospital, expresses, in a letter to the *Times*, his opinion that the malady is 'relapsing fever,' which, under different designations, has been well known in Britain and Ireland for nearly two centuries, which constituted a great part of the Irish epidemic of 1847, and which about the same time was very prevalent in various parts of Germany. The mortality from relapsing fever is not great, only three per cent., but Dr. Murchison shows that all epidemics of such fevers have co-existed with typhus, in which the mortality is twenty per cent. The public, he says, need be under little apprehension as to the importation of the Russian epidemic into England. The more formidable of the two diseases composing it is here already, and has, for the last three years been very prevalent among the poor of London."—*St. Petersburg Telegram to the London Times.*

BERLIN, April 7, 1865.

Authentic intelligence touching the Russian epidemic, states that three several maladies exist at the same time in St. Petersburg. In October last, *meningitis spinalis* appeared at St. Petersburg. This is a spasmodic affection of the brain and spinal cord, by which children are chiefly attacked; the mortality was from twenty to fifty per cent. In November, typhus was added to the first mentioned disease, occurring sporadically at first, and gradually developed into a malignant species of *febris recurrens.* The fever lasts a week at a time, the several attacks being separated by intervals as long. During these intervals the health is apparently so good that people have been dismissed from hospital who died soon after. A special committee has been formed, under Gov.-Gen. Suwaroff, to look after those apparently cured. On a second or third attack there is a general collapse, decomposition of blood, and paralysis. Quinine and stimulants have no effect. The deaths, at first but twenty, have risen to forty per cent. The spleen and liver are much affected. In many cases epidemical inflammation of the spleen, or *postula maligna,* has been observed. Quite recently the Siberian plague has broken out also; of this, seventy per cent. die within a few hours. A strong disposition to vomit, which cannot be satisfied, a swelling of the belly, pestilential carbuncles, and dark color of the skin, are its unmistakable symptoms. It is the "black death." St. Petersburg papers deny the existence of the plague in the capital, but the official *Northern Post* states it to have broken out at Szaniewo, in the Waldai Hills, and the description given in the St. Petersburg official *Medical News* and *Exchange News,* in which the dilatation of the pupils is especially dwelt upon, shows the malady, in its present stage, greatly to resemble the plague. In many cases, indeed, it is difficult to distinguish plague from *febris recurrens,* at the time when typhoid epidemics are abroad. The disease is apparently on the decrease. Dr. Erichson, surgeon to the Emperor Nicholas, aged seventy-five, died while attending hospital. In Poland, also, an epidemic has broken out. One case at Kole, near Warsaw, is represented in the *Warsaw Gazette* as *meningitis spinalis.* Out of five thousand inhabitants in that town, there are thirty-six sick and fifteen dead. In Eastern Prussia, there are many cases of *meningitis* near Dantzic.—*Correspondence of the London Times—Chicago Medical Examiner.*

